# Prevalence of Depressive Symptoms Among an Undergraduate Health Sciences Student Population: A Cross-Sectional Study

**DOI:** 10.7759/cureus.43117

**Published:** 2023-08-08

**Authors:** Saniya Jumani, Abdisamad Osoble, Tuba Ahmed, Tandis Rastegarlari, Mariam Hassan, Jayadevan Sreedharan

**Affiliations:** 1 Internal Medicine, Westford University College, Dubai, ARE; 2 Internal Medicine, Gulf Medical University, Ajman, ARE; 3 Emergency Medicine, Albany Medical Center, Albany, USA; 4 Pediatrics, University of Texas Medical Branch, Galveston, USA; 5 Internal Medicine, Texas Tech University Health Sciences Center El Paso, El Paso, USA; 6 Community Medicine, Gulf Medical University, Ajman, ARE

**Keywords:** undergraduate students, community health sciences, medical screening, major depressive disorder, depressive symptom, public mental health, depression prevention

## Abstract

Purpose: Undergraduate health sciences students are irrefutably liable to intrapersonal tension that may provoke the almost imperceptible onset and incremental expansion of depressive symptoms. Mental health is often a deplorably neglected topic despite posing as a catalyst in many students’ academic demise. Thus, the primary objective of this paper is to provide an insight into a multitude of variables that foster depressive symptoms. In doing so, the scope of subclinical depression that could be hindering a student's academic performance shall be illuminated.

Methodology: A cross-sectional study was conducted among health sciences students comprising both genders, any nationality, students 18 years of age or older, and students within their first three years of undergraduate study. The well-established Patient Health Questionnaire-9 was distributed along with a non-standardized questionnaire that inquires about additional risk factors. The chi-square test method was used to associate the dependent and independent variables, and statistical significance was done at p-value ≤ 0.05.

Findings: It was observed that 34.8% of participants suffer depressive symptoms. Participants’ sex and marital status, among many other factors, like age, program and year of study, are found to be statistically insignificant. Conversely, nationality, university-related workload, smoking, alcohol intake and more are noted to be significantly associated with the development of depressive symptoms.

Originality: This study is an original work done by the authors to investigate the prevalence of depressive symptoms among undergraduate health sciences students. The non-standardized questionnaire employed has been reviewed to ensure that it is without discrimination of any gender or biased towards any stakeholders.

## Introduction

The World Health Organization (WHO) unveils that depression, a common psychological disorder, affects more than 300 million individuals of all ages worldwide [1]. It presents with “loss of interest or pleasure, decreased energy, feelings of guilt or low self-worth, disturbed sleep or appetite, and poor concentration” [2].

The predisposing factors are innumerous, and they include genetic vulnerability, childhood adversity (neglect and abuse), and stressful life events, which all contribute to the development of depression itself. Lifestyle risks such as obesity, sedentary behavior, and smoking predispose to chronic general medical illnesses which also may also lead to mental, emotional and psychological turmoil and an increased likelihood of personal demise. Despite evidence-based guidelines for the treatment of depression being widely recognized, overall recorded improvement is low [3]. Undergraduate school, in particular, is a period of exposure to paramount stress, and the end result is catastrophic when depression runs unchecked and untreated. This, regrettably, leads to an increased rate of suicide. Therefore, the mental well-being of health sciences students should be of utmost importance, as they are the advancing future physicians of every society [4-8].

These concepts substantiate the notion of depression posing as a universal public health problem. It also attests to the need for unified efforts with regard to management and treatment [8]. In 2010, depression was ranked as the third leading cause of Disability Adjusted Life Years (DALY) in the Middle East/North Africa (MENA) region [8]. With that being said, the quantity of research investigating depression on a regional scale for the MENA region is relatively slim [8].

Firstly, the association between a student's living conditions and depressive symptoms is pondered upon. Living condition comprises living with family, a roommate or alone. Dependency of an individual on another and subconsciously knowing that their livelihood depends on them, for example, can instigate the occurrence of depressive symptoms. During periods of stress, "insecurely attached persons" are more likely to undergo drastic mood changes and may find fewer ways to cope with them [9]. These people may have a negative self-image or deem themselves less worthy, which contributes to depression [10].

Financial stress is yet another major contributory factor [11]. The prevalence of depressive symptoms between students who can pay for their tuition, those with partial or grant loans, and full loans has been compared. Financial vulnerability is found to further exacerbate depression in students from low-income families [11]. A meta-analysis of 60 students divulges that individuals “in the lowest socioeconomic quintile had 1.81 the probability of depression compared with those in the highest socioeconomic quintile” [12]. Therefore, a poorer socioeconomic status is significantly associated with a higher percentage of depressive symptoms.

Similarly, the pressure to overcome workload demands or a fall in academic performance is seen to evoke depressive symptoms, or vice versa [13]. This becomes particularly evident when there is an increase in the intensity level of the material being studied. A study executed in the United Kingdom concluded that depressive symptoms and emotional distress can have a significant effect on mid-course examination performance [13].

The excessive intake of alcoholic beverages is not only detrimental to one’s health but has been proven to cause social and economic burden within societies [14]. Alcohol dependence demonstrates a causal relationship with a range of mental and behavioral disorders, as elucidated by the WHO [14]. It is reported that about two-thirds of individuals who overindulge in alcohol consumption profusely endure feelings of sorrow and anxiety [15]. The repercussions of illicit drug use are also abrasive for social and economic development. A meta-analysis concerning the relation between depression and substance abuse has corroborated the idea that depression is consistently associated with “measures of cocaine-, alcohol-, and general drug use” [16].

Moreover, a mood disorder is an ambiguous phrase utilized by health professionals to group a class of mental health conditions that are related to depression and bipolar disorder [17]. A mood disorder entails an interplay of genetic and environmental factors and, hence, we delve into a multifactorial phenomenon. Evidence has been derived from twin and adoption studies to highlight that genetic factors do influence susceptibility to mood-related illnesses, leading to the familial aggregation of depressive symptoms [18].

A sleeping disorder, such as insomnia or excessive daytime sleepiness, preludes depression as a result of disturbances in monoamine activity [19]. Sleeping disorders form part of the clinical algorithm used to diagnose depression, and this is justified by the fact that non-depressed subjects with a family history of depression commonly have sleep abnormalities [19]. A coalesce of contemporary research proposes that sleep deprivation increases negative effects and gravely impacts mood, cognitive, and motor tasks [20]. Amongst a population of healthy adolescents, females are especially susceptible to this causal effect [21].

Lastly, a great deal of literature emphasizes the correlation between heightened consciousness of body image and depressive symptoms. A considerable risk factor in the commencement of mood disorders, like depression, is body image dissatisfaction [22]. Consequently, understanding predictors of negative body image is imperative for the management of depressive symptoms [22]. Through identification of these contributing variables, an individual might be more inclined to evade preconceptions of an ideal physique.

The diversity of triggers for symptoms suggestive of a possibly underlying depressive disorder is succinctly deliberated above. With that being said, the primary objective of this review is to appraise the various, yet more common, causes of depressive symptoms seen in a target population of undergraduate health sciences students. This allows for an approximation of the prevalence of depressive symptoms in a medical university while drawing attention to the students in a program whereby emotional and psychological assistance should be most sought out for. The secondary objectives involve assessment of the association between depressive symptoms and distinct sociodemographic factors, psychosocial determinants and lifestyle practices. This should help entice governing bodies of global universities to acknowledge the prospect of existing depressive symptoms within their own student body and unearth methods that could alleviate predisposing factors that threaten students' welfare and educational performance.

## Materials and methods

This cross-sectional study involved undergraduate students at Gulf Medical University in the United Arab Emirates (UAE). The following undergraduate programs were included: medicine (MBBS), dentistry (DMD), pharmacy (PharmD), physiotherapy (BPT), biomedical science (BBMS) and the allied health sciences which comprise anesthesia, medical imaging, medical laboratory and nursing (Table 1). Inclusion criteria encompass both male and female participants, students 18 years of age or older, individuals of any nationality and students within their first, second or third year of study (pre-clinical study years). Exclusion criteria included students younger than 18 years of age, fourth- or fifth-year health sciences students (clinical study years), and those who were unwilling to sign the consent form.

**Table 1:** Total Population of the Undergraduate Health Sciences Students MBBS: medicine, BBMS: biomedical science, DMD: dentistry, PharmD: pharmacy, BPT: physiotherapy, BHS: Bachelor's of Health Science

	Year 1	Year 2	Year 3	Total
MBBS	73	68	66	207
BBMS	35	21	-	56
DMD	51	48	38	137
PharmD	36	34	27	97
BPT	24	21	22	67
BHS	19	24	-	43
Total	238	216	153	607

The research was conducted over a period of 10 months through a self-administered questionnaire consisting of a globally recognized standardized survey and non-standardized segment. The first half is a multipurpose instrument for screening, diagnosing, monitoring and measuring the severity of depression, also referred to as the Patient Health Questionnaire-9 (PHQ-9) [23]. The PHQ-9 is accompanied by a Scoring Box to interpret the total score. The second half of the survey is a non-standardized questionnaire that was constructed by members of the research group to focus on other considerable aspects such as socioeconomic status and lifestyle patterns (Appendix Figures 1-3).

Approval was gained from the ethics committee of the medical university and deans of the respective colleges. The study was explained to the participants, a written consent was obtained from the willing, and all questions pertaining to the questionnaire were answered. Participants were given a thorough description of the purpose of the study, as well as its objectives. The researchers were present at the time of response to clarify any doubts. Questionnaires were collected on the same day.

Data analysis commenced using SPSS version 23 software (IBM Corp., Armonk, NY, USA). The number of students exhibiting depressive symptoms was expressed in frequencies and percentages. The Pearson Chi-Square Test was done to assess whether or not the risk factors of interest are truly associated with depressive symptoms. A p-value of ≤0.05 implies that a certain factor is considered statistically and significantly related to depression.

## Results

A total of 562 undergraduate health sciences students participated in the study, of which 74.4% were female and the remaining 25.6% were male (Table 2). An overwhelming majority (95%) of the students declared to be single. Most students reside with their family in the UAE (70.5%) and claim to be compatible with the person or people they live with (75.6%). A quarter of the undergraduate health sciences students are financially stressed (25.3%) and deem academic-related workload to be distressing (24.2%).

**Table 2:** The variables associated with a surge in depressive symptoms NS: Not Significant.  N/A: Not Applicable.

Variables	Groups	Evidence of Depressive Symptoms				p-values
		No		Yes		
		Frequency	%	Frequency	%	
Sex	Male	95	66.0	49	34.0	NS
	Female	271	64.8	147	35.2	
Marital status	Single	345	64.6	189	35.4	NS
	Married	21	75.0	7	25.0	
Living with family in the UAE	Yes	257	64.9	139	35.1	NS
	No	109	65.7	57	34.3	
Getting along with housemate(s)	Yes	281	66.9	139	33.1	≤ 0.01
	No	28	47.5	31	52.5	
	N/A	83 (14.8%)				
Worried about debt and/or lack of funds	Yes	73	51.4	69	48.6	≤ 0.001
	No	293	69.8	127	30.2	
Overwhelming workload or stopped enjoying other activities	Yes	70	51.1	67	48.9	≤ 0.001
	No	72	69.6	31	30.1	
	Sometimes	224	69.6	98	30.4	
Satisfaction with academic performance	Yes	184	74.5	63	25.5	≤ 0.001
	No	44	47.8	48	52.2	
	More often	103	62.8	61	37.2	
	Uncertain	35	59.3	24	40.7	
Alcohol consumption	Yes	16	41.0	23	59.0	≤ 0.001
	No	350	66.9	173	33.1	
Sought after recreational drug(s)	Yes	10	37.0	17	63.0	≤ 0.001
	No	356	66.5	179	33.5	
Family history of any mood disorder(s)	Yes	26	50.0	26	50.0	≤ 0.05
	No	340	66.7	170	33.3	
Coping with a sleeping disorder(s)	Yes	21	34.4	40	65.6	< 0.001
	No	345	68.9	156	31.1	
Satisfied with physique	Yes	220	69.6	96	30.4	≤ 0.05
	No	146	59.3	100	40.7	

Just less than half of the students (43.4%) responded ‘Yes’ when asked whether they are content with their academic achievements. In light of the region in which this study has been conducted, it is not surprising that many do not indulge in consumption of alcoholic beverages or partake in illicit drug use. Ninety-four percent and 95.2% of students denied consumption of psychiatric and recreational drugs, respectively. Approximately 90% of students do not have a personal or family history of mood disorders or abnormal sleeping habits. Lastly, 43.8% of the participants are not pleased with their physical appearance.

Table 2 reveals the frequency of variables associated with a potential surge in depressive symptoms, followed by the calculated p-value that is indicative of statistical significance. A positive correlation is denoted by a p-value less than or equal to 0.05 which is not seen with sex, marital status or living circumstance. On the contrary, the table exhibits a true relation between the remaining variables and evidence of depressive symptoms. The p-value ≤0.001, in particular, asserts a highly, statistically significant relationship between smoking and financial burden, for example.

## Discussion

The results show that the crude prevalence of depressive symptoms is 34.8%. This prevalence is higher compared to other studies conducted in the Middle East [24,25]. The participants’ sex and marital status, among many other factors, like age, program and year of study, are found to be statistically insignificant. Conversely, nationality, university-related workload, smoking, alcohol intake and more are noted to be significantly associated with the development of depressive symptoms. The present study underlines an overall greater propensity for depression. This could be explained by the difference in number of entrees and cultural demographic variations, for example. Additionally, medical studies can be more rigorous and impose a greater deal of mental stress unlike other programs.

A similar study was conducted in Al Ain, UAE to ascertain the prevalence of depressive symptoms and its socioeconomic determinants among university students. The study concluded that the prevalence of depressive symptoms among university students is approximately 22.2% and that age, rather than nationality, is more associated with depressive symptoms [24]. While the prevalence of depressive symptoms is lower in comparison, it is one that cannot be neglected.

Another systematic review concludes that the prevalence of depressive symptoms among university students is 27.7% [25]. Yet again, this percentage is lower compared to the figure achieved in the current study and this inaccuracy can be explained by the small study population and lack of an accurate psychological assessment. The authors recommend that future research studies evaluate clinical students (fourth and fifth year of study) as well as expand the investigation to health sciences students of other notable universities in the Middle Eastern region.

To conclude, depressive symptoms are widespread and similar trends have been observed in previous studies. A comprehensive screen must be devised to diagnose students that are inclined to progress in their symptomatology towards a diagnosis of major depressive disorder early on. Immediate treatment and support should be provided to health sciences students in the form of counseling and simultaneously denouncing stigmatization of mental health-related concerns. Students that are initially reluctant to seek help or have difficulty confiding in another during periods of distress are being unknowingly bolstered.

## Conclusions

The present cross-sectional study deduces that 34.8% of undergraduate health sciences students bear evidence of depressive symptoms. This statistic is especially alarming as we are now aware of having a pool of students that are unquestionably at risk of psychological deterioration. Several variables have been found to contribute towards poor mental health, including financial status, academic performance, workload, smoking, alcohol consumption, recreational drug use, family history of a mood disorder(s), sleeping disorder(s) and physique - all of which are statistically and significantly related to a rise in depressive symptoms.

Identification of the primary contributing factors and enhancing the education system to become more student-friendly are key methods in tackling the current prevalence of depressive symptoms. Educational reform could mean minimizing the competitiveness of medical schools, restructuring the grading criteria or methods of evaluating students. Undergraduate schools can, for example, endorse the importance of sleep and effective sleep hygiene, as it is crucial in this stage of life, as well as condemn the internalization of a body image crisis that could very well underpin depressive symptoms.

## Figures and Tables

**Figure 1:**
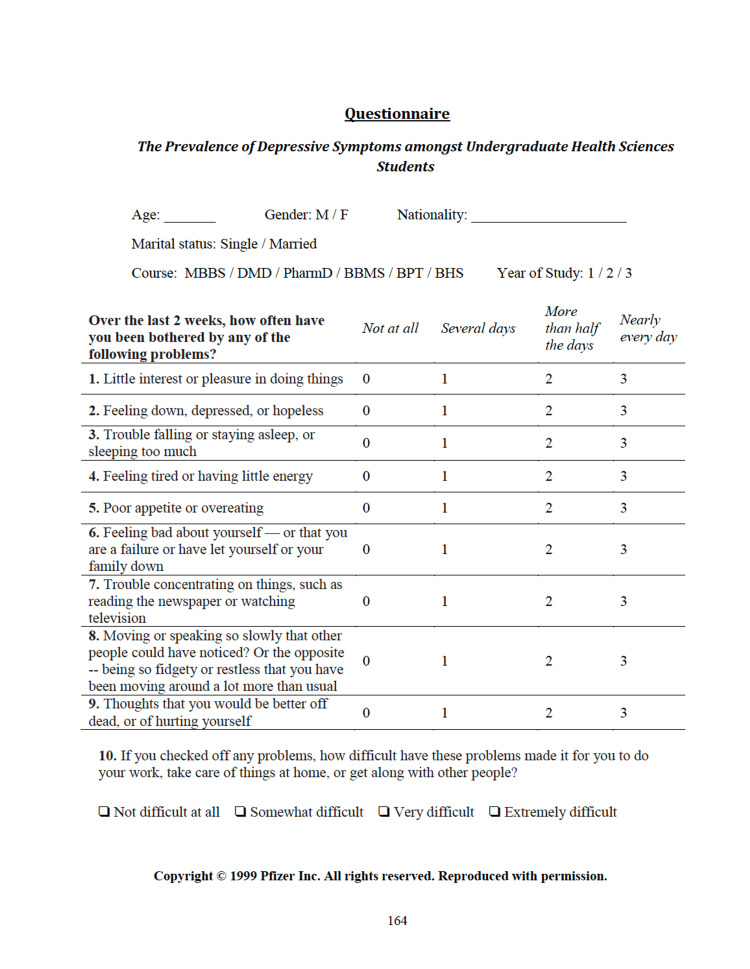
Questionnaire Part 1

**Figure 2:**
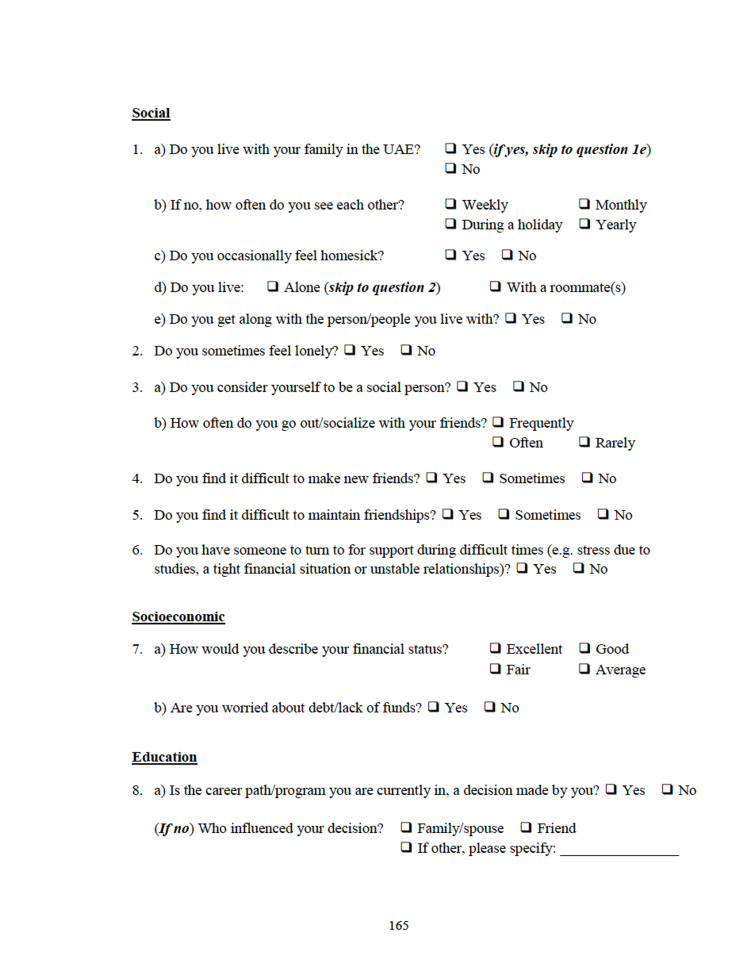
Questionnaire Part 2

**Figure 3:**
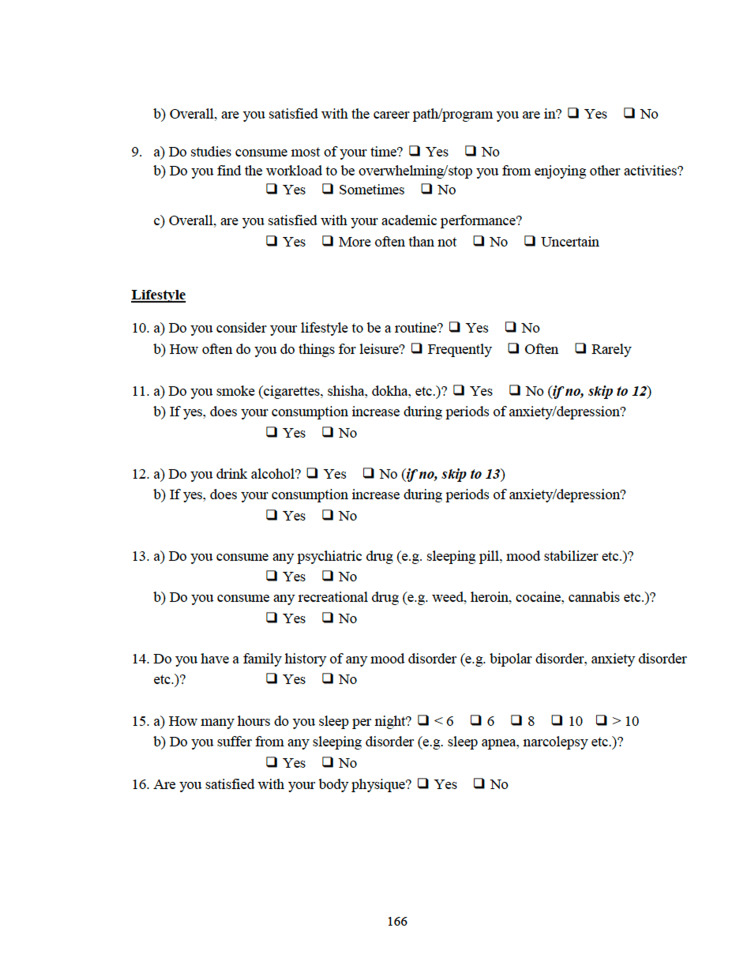
Questionnaire Part 3
